# Plasma presepsin level is an early diagnostic marker of severe febrile neutropenia in hematologic malignancy patients

**DOI:** 10.1186/s12879-016-2116-8

**Published:** 2017-01-05

**Authors:** Yusuke Koizumi, Kaoru Shimizu, Masayo Shigeta, Takafumi Okuno, Hitoshi Minamiguchi, Katsuyuki Kito, Keiko Hodohara, Yuka Yamagishi, Akira Andoh, Yoshihide Fujiyama, Hiroshige Mikamo

**Affiliations:** 1 Department of Clinical Infectious Diseases, Aichi Medical University, 1-1 Yazakokarimata, Nagakute, Aichi 480-1195 Japan; 2 Department of Gastroenterology and Hematology, Shiga University of Medical Science, Otsu, 520-2192 Japan; 3 Department of Laboratory Medicine, Shiga University of Medical Science, Otsu, 520-2192 Japan

**Keywords:** Presepsin(soluble CD14-ST), Febrile neutropenia, Bacteremia, Chemotherapy, CD14

## Abstract

**Background:**

Febrile neutropenia (FN) is a common infectious complication in chemotherapy. The mortality of FN is higher in hematologic malignancy patients, and early diagnostic marker is needed. Presepsin is a prompt and specific marker for bacterial sepsis, but its efficacy in severe febrile neutropenia (FN) is not well confirmed. We tried to clarify whether it is a useful maker for early diagnosis of FN in patients during massive chemotherapy.

**Methods:**

We measured plasma presepsin levels every 2–3 day in FN cases and evaluated its change during the course of massive chemotherapy. The patients had hematologic malignancy or bone marrow failure, and in all cases, neutropenia was severe during the episode. The baseline levels, onset levels, increase rate at FN onset, and onset / baseline ratio were evaluated for their efficacy of early FN diagnosis.

**Results:**

Eleven episodes of bacteremia (six gram negatives and five gram positives) in severe neutropenia were analyzed in detail. While plasma presepsin level was strongly associated to the CRP level (*r* = 0.61, *p* < 0.01), it was not associated with the absolute WBC count (*r* = −0.19, *p* = 0.19), absolute neutrophil count (*r* = −0.11, *p* = 0.41) or absolute monocyte count (*r* = −0.12, *p* = 0.40). The average of onset presepsin level was 638 ± 437 pg/mL and the cutoff value (314 pg/mL) has detected FN onset in 9 of 11 cases. The two cases undetected by presepsin were both *Bacillus* species bacteremia.

**Conclusions:**

Plasma presepsin level is a reliable marker of FN even in massive chemotherapy with very low white blood cell counts. Closer monitoring of this molecule could be a help for early diagnosis in FN. But bacteremia caused by *Bacillus species* was an exception in our study.

**Electronic supplementary material:**

The online version of this article (doi:10.1186/s12879-016-2116-8) contains supplementary material, which is available to authorized users.

## Background

Patients with hematologic malignancies often suffer from severe infections. The malignancies themselves are disorders of immune systems, and chemotherapy further causes febrile neutropenia (FN). Some patients die of infection in spite of immediate antimicrobial treatment. In acute myeloid leukemia, for example, the incidence of chemotherapy associated infectious toxicities is as high as 64%, and the mortality is higher than 10% [1]. There have been many approaches, including biomarkers, to improve the prognosis of FN. But still, it is difficult to predict or to detect those malicious events in the earliest time.

Presepsin (soluble CD14 subtype) is a novel biomarker of sepsis. It is useful in early diagnosis, and it can also be a prognostic marker of severe sepsis [2–6].

While this molecule is a prompt and specific marker for bacterial infections, its reliability in FN is not yet confirmed. Especially, massive chempotherapy causes severe cytopenia including CD14 expressing neutrophils or monocytes. Because presepsin derives from CD14 [7], severe neutropenia can cause false-negativity of this molecule.

Thus, we conducted a simple study of plasma presepsin measurements to find answer to two practical questions;

1. Does presepsin rise in bacteremia cases even in the setting of severe neutropenia?
2. Is plasma presepsin level a useful maker for early diagnosis of FN?

## Methods

### Study population

This study was conducted in Shiga University of Medical Science Hospital, from May 2014 to October 2014.

During the period, plasma samples were collected from hematologic malignancy and bone marrow failure patients under chemotherapy. The patients received conventional induction chemotherapy or hematopoietic stem cell transplantation (HSCT) and all of them experienced severe neutropenia.

We evaluated the plasma presepsin levels in febrile neutropenia group (FN group). And we compared the values with those of control cases, afebrile neutropenia group (AFN group).

We defined two groups as follows;

1. FN group patients are those diagnosed of bacteremia during neutropenic period (WBC count less than 1,000/μL or neutrophil count less than 500 /μL). Pathogens were identified in blood cultures and patients were treated with appropriate antimicrobial therapy.
2. AFN group patients were those who had no febrile episodes for 3 weeks after chemotherapy. They received no antimicrobials during the course.

### Plasma presepsin level measurement

We made regular blood examination every 2–3 day (approximately on days 1,3,5,8,10,12 and 15) of chemotherapy. After centrifugation within 2 h after phlebotomy, the plasma was preserved in −80 °C till measurement.

Plasma presepsin level was measured by a rapid chemiluminescent enzyme immunoassay on the fully automated PATHFAST® immunoanalyzer (Mitsubishi Chemical Medience Corporation, Tokyo, Japan) [8]. We defined the cut-off value 314 pg/mL according to manufacturer’s instruction.

### Evaluation

We evaluated serial change in plasma presepsin levels at regular intervals.

Specifically, the value obtained on day −1 to 1 of chemotherapy was defined as baseline level. We defined the first day of fever onset (≥38.0 °C) as the “onset day”. Blood culture was routinely obtained within ±1 day of fever onset.

The “onset plasma presepsin level (onset level)” was defined as the result on the day of fever and blood culture positivity.

And the presepsin increase rate (IR) was defined as

$$ \mathrm{I}\mathrm{R}\left(\%\right)=\frac{\left\{\left(\mathrm{onset}\ \mathrm{level}\right)\hbox{-} \left(\mathrm{previous}\ \mathrm{level}\right)\right\}/\left(\mathrm{previous}\ \mathrm{level}\right) \times 100\ }{\mathrm{Blood}\ \mathrm{examination}\ \mathrm{interval}} $$

The onset levels within one episode, presepsin IR around the day of febrile neutropenia onset, and onset / baseline ratio were evaluated.

### Statistical analysis

We performed Student’s *t*-test for comparisons of two independent groups of sampled data. The data were expressed as mean ± standard deviation. *P* values of <0.05 were considered evidence of a significant difference.

The linear dependence between the two variables was assessed by Pearson’s product–moment coefficient. R and Rho values ≥0.7 were considered to define a strong correlation between variables, respectively. And R and Rho ranging between 0.69 and 0.5 and 0.49 to 0.3 were considered to define moderate and low correlation, respectively.

We analyzed data using the statistical software JMP® 10 (SAS Institute Inc., Cary, NC, USA).

## Results

### Patient background (Table 1)

**Table 1:** Patient characteristics

Comorbidities	Acute myeloid leukemia	3
	multiple myeloma	2
	malignant lymphoma	2
	acute lymphoid leukemia	1
	myelodysplastic syndrome	1
	aplastic anemia	1
chemotheapy	induction therapy	6
	conditioning regimen hematopoietic stem cell transplantation	3
	consolidation therapy	1
Nadir WBC count during the chemotherapy	0–100/μL	9
	101–500/μL	1
	501- /μL	0
focus of infection^a^	primary bacteremia	9
	severe pneumonia	1
	skin & soft tissue infection	1
pathogens identified in blood cultures		
Gram negatives		6
*K.pneumoniae*		3
*E.coli*		1
*E.aerogenes*		1
*B.fragilis*		1
Gram positives		5
*S.epidermidis*		1
*S.hominis*		1
*B.cereus*		1
*Bacillus sp.*		1
*E.gallinarum*		1
days of the infection onset^b^		11.9 [3–18]
average number of the samples examined for presepsin		6.5 [4–9]

^a^focus denotes the entry site of bacteremia

^b^day 1 is defined as the day when chemotherapy regimen starts

Table 1 shows the patient characteristics.

There were five myeloid diseases, including three acute myeloid leukemia (AML), 1 myelodysplastic syndrome (MDS) RAEB2, and 1 severe aplastic anemia (AA).

Other five cases were lymphoid diseases, including two malignant lymphoma (ML), two multiple myeloma (MM), and one acute lymphoid leukemia (ALL).

All the cases were on chemotherapy; six on induction therapy, three on myeloablative conditioning regimen for hematopoietic stem cell transplantation, and one on consolidation therapy. All the regimens had potentials of severe myelosuppression.

The Nadir WBC count during the chemotherapy was 0–100/μL in nine out of ten cases. There were no cases with severe renal failure.

### Episodes of febrile neutropenia (Table 1)

Eleven episodes of febrile neutropenia were observed in ten patients; 9 with primary bacteremia, 1 with severe pneumonia, and 1 with skin & soft tissue infection.

Pathogens identified in blood cultures were, six gram negative bacteria (3 *Klebsiella pneumoniae*, 1 *Escherichia coli*, 1 *Enterobacter aerogenes*, and 1 *Bacteroides fragilis*) and five gram positive bacteria (1 *Staphylococcus epidermidis*, 1 *Staphylococcus hominis*, 1 *Bacillus cereus*, 1 *Bacillus sp.*, and 1 *Enterococcus gallinarum*).

The onset of infection was on the day 11.9 (average, range [3–18]) of chemotherapy, and 6.5 (average, range [4–9]) samples per one case were examined for plasma presepsin level.

All FN patients survived bacteremia after receiving appropriate antimicrobial agents, though one case had severe acute respiratory distress syndrome requiring intensive care.

### Change of plasma presepsin levels in FN cases

**Fig. 1:**
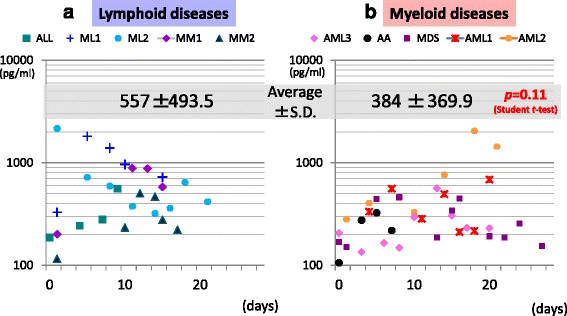
Plasma presepsin level change over time and comorbidities. Changes in plasma presepsin level over time in lymphoid malignancy cases (**a**) and myeloid malignancy cases (**b**) are shown. Horizontal axis shows the day of chemotherapy. The average of plasma presepsin level was lower in myeloid malignancies. Note that most of the presepsin values are above 100 pg/mL, suggesting that it maintains certain levels even in severe neutropenic state

Figure 1 shows the changes in plasma presepsin level over time during a chemotherapy course. All the samples measured in this study are plotted in the figure.

The average of presepsin levels in myeloid diseases (384 ± 369.9 pg/mL was slightly lower than that of lymphoid diseases (384 ± 369.9 557 ± 493.5pg/mL, *p* = 0.11, Student’s t-test).

There was no significant decrease within the first week of chemotherapy, which usually causes severe neutropenia.

The lowest value was 82.6 pg/mL scored in case 8, a multiple myeloma case, on day 8 of HSCT conditioning regimen. On that day, the patient was afebrile with WBC count of 100/μL, but 4 days later, he developed bacteremia with presepsin level of 503 pg/mL. The detailed trend of presepsin and CRP levels are shown in Additional file 1: Table S1.

### Plasma presepsin levels and other parameters

**Fig. 2:**
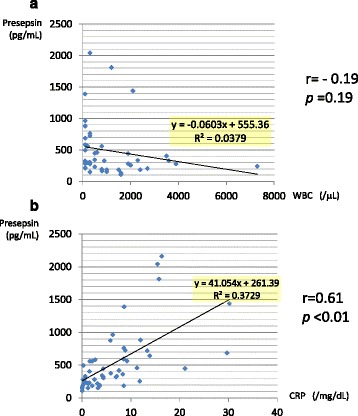
Plasma presepsin levels and other parameters measured at the same time point. **a** Plasma presepsin levels white blood cell count. White blood cell count and plasma presepsin levels were plotted. Plasma presepsin levels were not significantly associated to the absolute white blood cell count(*r* = −0.19, *p* = 0.19). Plasma presepsin level was not associated with neutrophil count, or monocyte count, either (data not shown). **b** Plasma presepsin levels and C-reactive protein (CRP) levels. CRP levels and plasma presepsin levels were plotted. Plasma presepsin levels were strongly associated to the CRP levels. (*r* = 0.61, *p* < 0.01)

Figure 2a shows the relationship between plasma presepsin level and white blood cell count (WBC).

Plasma presepsin level was not significantly associated with the absolute WBC count (*r* = −0.19, *p* = 0.19).

The median plasma presepsin level was 456.5 pg/mL in low WBC count below 100 /μL.

The plasma presepsin level was not associated with absolute neutrophil count (*r* = −0.11, *p* = 0.41) or absolute monocyte count (*r* = −0.12, *p* = 0.40), either. Meanwhile, plasma presepsin level was strongly associated to the CRP level (*r* = 0.61, *p* < 0.01, Fig. 2b.)

We also examined the association between presepsin levels and renal function.

The plasma presepsin level was not associated with serum creatitine level (*r* = 0.08, *p* = 0.55), or estimated glomerular filtration rate (eGFR) (*r* = 0.21, *p* = 0.12).

### Baseline, onset plasma presepsin levels and increase rates in FN cases

Onset levels, increase rates (IR) and onset / baseline ratio of presepsin are shown in Table 2

**Table 2:** Plasma onset presepsin levels and increase rates in 11 febrile neutropenia cases with identified pathogens (FN group) and 4 afebrile neutropenia cases (AFN group)

**FN group**											
Case	Commobidity	Pathogen identified in blood culture	Baseline level	Onset level	Previous level	blood examination interval^a^	Increase rate	Onset /Baseline ratio	Blood cell counts at onset day (/μL)		Remarks
			(pg/mL)	(pg/mL)	(pg/mL)	(day)			WBC	neutrophils	
*1*	AML	*E.coli*	280	760	329	4	33%	2.71	300	3	pneumonia(ARDS) ▪ ICU stay
*2*	AML	*K.pneumoniae*	206	562	296	3	30%	2.73	200	0	bacteremia
*3*	ALL	*K.pneumoniae*	187	558	279	2	50%	2.98	200	0	bacteremia
*4*	MDS	*K.pneumoniae*	169	341	186	2	42%	2.02	200	0	bacteremia
*5*	ML	*E.aerogenes*	330	1810	330	4	112%	5.48	100	0	bacteremia
											bacteremia
*6*	MM	*B.fragilis*	201	884	201	10	33%	4.4	<100	0	bacteremia + diverticulitis
*Average (gram negatives)*			229 ± 62.4	819 ± 520			50%	3.39 ± 1.29			
*7*	ML	*E.gallinarum*	320	644	361	2	39%	2.01	<100	0	bacteremia + HPS
*8*	MM	*S.epidermidis*	116	503	234	2	57%	4.34	<100	0	bacteremia + CRBSI
*9*	MDS	*S.hominis*	169	460	151	4	51%	2.72	<100	0	bacteremia
*10*	AA	*Bacillus sp.*	106	275	106	4	40%	2.59	300	250	bacteremia + CRBSI
*11*	AML	*B.cereus*	333	217	211	2	1%	0.65	<100	0	bacteremia + meningitis
*Average (gram positives)*			209 ± 110.2	420 ± 174			38%	2.46 ± 1.33			
*Average (FN group)*			220 ± 83.1	638 ± 437			44%	2.97 ± 1.33			
AFN group											
Case	Commobidity	Pathogen identified in blood culture	Baseline level	Onset level^b^	Previous level	Blood examination interval^a^	Increase rate	Onset /Baseline ratio	Blood cell counts at onset day (/μL)		Remarks
			(pg/mL)	(pg/mL)	(pg/mL)	(day)			WBC	neutrophils	
*12*	AML		174	223	172	2	15%	1.28	400	48	
*13*	AML		138	298	137	2	59%	2.16	100	0	
*14*	MM		49	188	89	5	22%	3.84	100	0	
*15*	ML		120	247	136	7	12%	2.06	100	0	
*Average (AFN group)*			120 ± 52.5	239 ± 46.2			27%	2.33 ± 1.08			

Abbreviations: *ARDS* acute respiratory distress syndrome, *CRBSI* catheter related blood stream infection, *HPS* hemophagocytic syndrome, *WBC* white blood cell

^a^blood examination interval denotes the interval between peak level and previous level (day)

^b^in the AFN group, onset level denotes the highest value within one chemotherapy course

The average baseline level was 220 ± 83.1 (range 106–333) pg/mL and average onset level was 638 ± 437 (range 217–1810) pg/mL.

In the episodes with gram negative bacteremia, onset levels were elevated beyond cut-off level in all cases (average 819 ± 520 pg/mL, range 341–1810 pg/mL).

In the episodes with gram positive bacteremia, onset levels were elevated in 3 out of 5 cases (average 420 ± 97.0 pg/mL, range 217–644 pg/mL). The onset presepsin levels are slightly higher in gram negative bacteremia than gram positive bactermia (*p* = 0.14, Student’s *t*-test). Two cases of *Bacillus* bacteremia (case 10,11) showed no elevation of onset presepsin levels at the onset time.

The average increase rate (IR) was 44 (range 1–112) % and, nine out of 11 bacteremic FN cases showed IR of more than 30%.

When we apply onset value ≥ 314 pg/mL and/or IR larger than 30% as positive findings, we could detect 10 out of 11 febrile neutropenia cases.

Or, onset / baseline ratio above two has detected 10 out of 11 cases.

### Presepsin levels in representative cases

We show representative FN cases, demonstrating the efficacy of plasma presepsin levels in early diagnosis of septicemia.

**Fig. 3:**
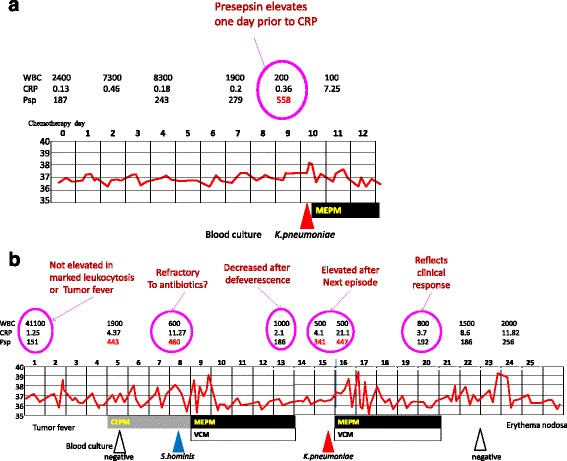
Plasma presepsin levels in representative cases. **a** Case 3; 48 year old male with B-ALL, undergoing “JALSG Ph(−) B-ALL 213 consolidation” chemotherapy. He experienced *K.pneumoniae* bacteremia on day 10. WBC count was 200/mL and plasma presepsin level was already elevated one day prior FN onset. CRP was not elevated at this time. **b** Case 4; 68 year old female with MDS and Sweet disease, undergoing induction chemotherapy (MEC-GO). Plasma presepsin level was not elevated in spite of marked leukocytosis (day1) or non-infectious fever caused by erythema nodosa (day24). It was elevated at the onset of *S.hominis* bacteremia (day8), and at *K.pneumoniae* bacteremia (day15)

Case 3 (Fig. 3a) shows typical clinical course of gram negative bacteremia in a case with B cell lineage acute lymphoid leukemia. The onset of febrile neutropenia was on day 10 of consolidation therapy. On day 9, presepsin was elevated to 558 pg/mL while CRP was within normal range at the same time. The blood culture on day 10 has detected *K.pneumoniae*.

Case 4 (Fig. 3b) shows the dynamics of presepsin levels in MDS case. On day 1 of chemotherapy, presepsin level was not elevated in spite of marked leukocytosis and tumor fever. On day 5, presepsin elevated and cefepime was started, and on day 8, the patient developed methicillin resistant *S.hominis *bacteremia. Probably the elevation of presepsin suggested uncontrolled bacteremia with the organism resistant to cefepime. Meropenem and vancomycin was started, and soon the fever has subsided, accompanied by presepsin level decrease. One day after cessation of antibiotics, the case developed high fever due to *K. pneumoniae* bacteremia. Presepsin was measured 6 h prior to fever and chills, and was elevated again, suggesting the ability to detect infection even in an asymptomatic state. After appropriate antibiotic treatment, presepsin level was normalized again. Meanwhile, neutrophil count was below 600/μL. A few days after resolution, the temperature has risen again with erythema nodosa,but presepsin level was within normal range.

In cases of *Bacillus* sp., plasma presepsin level was not elevated at the onset of febrile neutropenia. In both cases, presepsin level was elevated 2 days after the onset. Case 10 is the aplastic anemia patient in HSCT. He suffered from Bacillus bacteremia as blood stream infection. The onset of FN was on day 3 of chemotherapy. The presepsin level was within normal range on the onset day, but 2 days later, it has increased to 325 pg/mL. Case 11 was AML patient on induction chemotherapy. On day 18, she developed bacteremia and severe meningitis. But presepsin level was 217 pg/mL. Two days later, it showed an increase to 685 pg/mL.

### Presepsin levels of AFN cases in comparison to FN cases

Table 2 shows the result of parameters in AFN cases. As for AFN cases, the average of baseline plasma presepsin levels was 120 ± 52.5 pg/mL. There was no drastic decrease during the severe neutropenic period and none of the values was above the cut off level.

The average of all examined presepsin levels in AFN group (*n* = 25) was 162 ± 61.7 pg/mL, and it was significantly lower than that of FN group (*n* = 69) (451 ± 304 pg/mL, *p* = 0.0012).

The highest levels in AFN cases were also compared with onset plasma presepsin levels of FN group.

The average of the highest presepsin levels was 239 ± 46.2 pg/mL in AFN cases. It was slightly lower than in FN cases (638 ± 437 pg/mL, *p* = 0.099, Student’s *t*-test).

## Discussion

Presepsin is a one of the subtypes of soluble N-terminal fragment of CD14 protein with molecular weight of 13 kDa. It is the receptor for lipopolysaccharide (LPS) / LPS –binding protein complexes [2] and is released in the early phase of infection. The molecule rises as early as within 2 h of inflammation onset, which is even earlier than procalcitonin, C-reactive protein (CRP) or interleukin-6 [2, 9]. Another fascinating aspect of prespsin is its high specificity for bacterial infections. Because its precursor CD14, expressed on the surface of phagocytes, is internalized during bacterial phagocytosis, the processed subtype presepsin has strong association with bacterial infections. Nowadays, it is well known that presepsin is a useful early diagnostic marker of sepsis. And there are reports showing its superiority to CRP in differentiating bacteremic SIRS from that of other causes [10]. Its diagnostic efficacy has been proved in various fields such as surgery, burn, neonates, and central nervous system [11–14]. However, the reliability of presepsin in febrile neutropenia has not been well demonstrated so far. Probably the fact that CD14 is expressed in neutrophils or monocytes, might pose a question against its quality, especially in a severe neutropenia after massive chemotherapy.

Our study is one of the first reports showing dynamics of plasma presepsin levels in FN cases. And we proved that 1) presepsin maintains a proper level in severe neutropenia cases, and 2) presepsin is a useful marker for early diagnosis of FN.

The most important finding in this study is that presepsin level was not associated with WBC, neutrophil, or monocyte count. The baseline and the lowest plasma presepsin levels were not extremely low as expected even in neutropenia settings. Giavarina et. al. [15] studied the presepsin levels of healthy adult controls, concluding that the reference limits for the presepsin were 55–184 pg/mL (90% confidence intervals, CI, were 45 to 58 and 161 to 214, respectively). The lowest value in our study was 48.7 pg/mL (AFN group), which is not so low compared with those reference data. Thus we can say that presepsin maintains a proper level even in severe neutropenia cases. It might be explained by the recent finding that monocyte, rather than neutrophil, is the dominant producer of presepsin [16]. Production from tissue macrophages or resident monocytes might play a role in maintaining the plasma presepsin level.

Another result is that plasma presepsin level was significantly elevated at the early phase of most bacteremia episodes. The data have shown a clear relationship between presepsin level and CRP, suggesting its sensitivity and validity in evaluation of infection. Especially in gram negative bacteremia, it was higher than the normal range in all the cases. Various cutoff levels of presepsin has been proposed to detect bacterial infection, but in those reports, the background of the cases (Intensive care units, emergency room, or general medicine), and goals of the studies (discrimination of bacterial/non bacterial infection, or early detection of sepsis) are different. We simply applied the cutoff level as written in the manufacturer’s instruction. The cutoff value of 314 pg/mL was lower than some of the reports [2–6]. We aimed to improve sensitivity rather than specificity because our goal is to detect the FN onset in the early phase and to prevent overt sepsis. However, in the two FN bacteremia cases caused by *Bacillus* species, presepsin level stayed within the normal range at the onset day. We could say presepsin level is not appropriate to detect early phase of *Bacillus* bacteremia, though the reason cannot be readily explained. Probably it might be due to the low immunogenicity by this bacterium.

The size of this study was small, and retrospective design can be a limitation. Yet, the retrospective nature of this study has four advantages at least. First, we selected cases with definite diagnosis of bacteremia and severe complications. Second, we included only the cases with severe neutropenia, whose median WBC count at FN onset were 100 /μL. Third, none of the cases had severe renal dysfunction which interferes to the presepsin value [17]. Fourth, the measurement of presepsin levels was made regularly, not in spot, throughout a chemotherapy course and FN episode. So we could follow up the trend from early phase to resolution of bacteremia. Many of the reports deal with mass data yielded from hundreds of patients. But detailed understanding and interpretation of clinical course of every case is also important.

## Conclusion

In conclusion, plasma presepsin level is a reliable marker of FN even in extremely low WBC counts. In addition to absolute value, evaluation of increase rate can help early diagnosis of FN in both myeloid and lymphoid disorders. Closer monitoring of this molecule could prevent infection associated death in hematologic malignancy cases.

## Additional file

Additional file 1: Table S1. Plasma presepsin level change over time compared with CRP in FN group (cases 1–11) and AFN group (cases 12–15). (XLSX 13 kb)

## Abbreviations

AA: Aplastic anemia
AFN: Afebrile neutropenia
ALL: Acute lymphoid leukemia
AML: Acute myeloid leukemia
CRP: C reactive protein
FN: Febrile neutropenia
HSCT: Hematopoietic stem cell transplantation
IR: Increase rate
LPS: Lipopolysaccharide
MDS: Myelodysplastic syndrome
ML: Malignant lymphoma
MM: Multiple myeloma
RAEB: Refractory anemia with excessive blast
WBC: White blood cell
